# Unusual CT and MR Imaging Characteristics of Splenic Lymphoma

**DOI:** 10.1155/2011/298357

**Published:** 2011-12-22

**Authors:** Chhavi Kaushik, Nitin Relia, Kedar Jambhekar, Tarun Pandey

**Affiliations:** ^1^Department of Radiology, University of Arkansas for Medical Sciences, Little Rock, AR 72205, USA; ^2^Department of Internal Medicine, University of Arkansas for Medical Sciences, Little Rock, USA

## Abstract

Lymphoma is the most common malignancy of the spleen. The imaging features of splenic lymphoma are nonspecific and mostly lymphomas present as a diffusely enlarged spleen. Focal lesions are described but remain of low density or intensity on CT or MRI, respectively. We describe a histologically proven case of splenic lymphoma that showed an atypical hyperdense/hyperenhancing appearance on imaging suspicious for a vascular pathology. To the best of our knowledge and based on review of English literature, such an appearance of splenic lymphoma is extremely unusual and rare.

## 1. Introduction

Lymphoma is the most common tumor of the spleen and it may be primary or secondary to a diffuse lymphomatous process [1]. Splenic involvement in lymphoma is important in deciding the line of treatment and determines the overall prognosis. Splenic involvement by lymphoma has varied appearances including the spleen being normal that is microscopic involvement or splenomegaly with or without focal mass lesions [2, 3]. Focal involvement is typically hypodense on noncontrast CT and mildly hyperintense on T2-weighted MRI with poor postcontrast enhancement on both [2–4]. Our case is unique in that the splenic lesions of lymphoma were intensely enhancing on CT and MRI, which is not typically described or expected on imaging of splenic lymphoma.

## 2. Case Report

A 60-year-old Caucasian male presented to the ER with one-month history of weakness, fatigue, and left upper quadrant pain. Past medical history was significant for diabetes Mellitus, hypertension, and hyperlipidemia. Patient worked in a fertilizing chemical business with history of exposure to chemicals and pesticides over the last 2 decades. Physical examination was remarkable for pallor and splenomegaly extending 7-8 cm below costal margin. Laboratory analysis showed hemoglobin of 9 g/dL, hematocrit 28%, white count 4.0 × 10^3^/mm^3^, and platelet count of 70 × 10^3^/*μ*L. Peripheral blood smear did not show any abnormal lineage cells.

**Figure 1:**
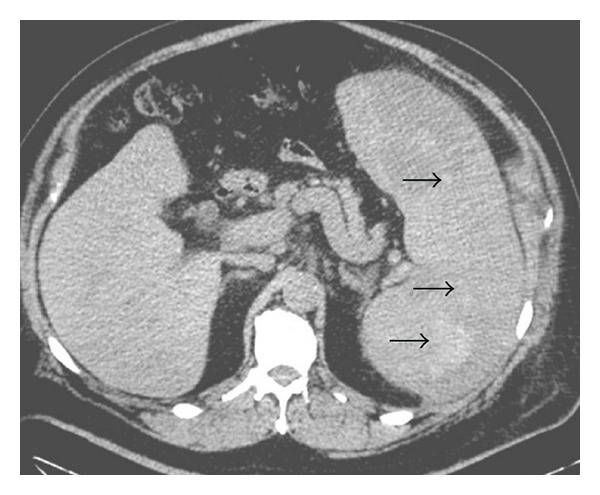
Axial noncontrast CT section through the abdomen showing splenomegaly with multiple hyperdense nodules in the splenic parenchyma (arrowheads).

**Figure 2:**
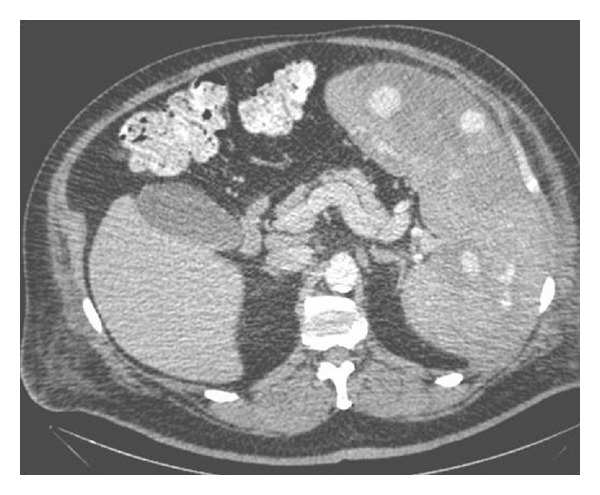
Lesions showing intense enhancement on post contrast CT images.

**Figure 3:**
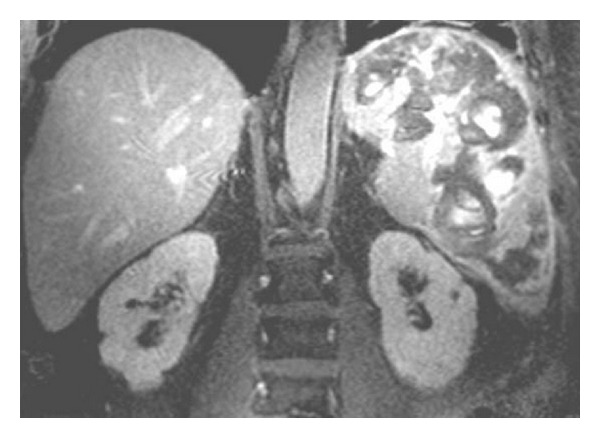
Coronal postcontrast MR image showing splenomegaly with multiple intensely enhancing masses.

Noncontrast CT abdomen showed massive splenomegaly with multiple hyperdense foci within the spleen (Figure 1, arrowheads) representing hemorrhage within the splenic parenchyma. Post contrast CT (Figure 2) revealed multifocal intensely enhancing areas in the splenic parenchyma suggesting the highly vascular nature of these masses. Differential diagnoses based on these findings included atypical hemangioma, splenic peliosis, and angiosarcoma [4, 5]. MRI revealed massive splenomegaly with multifocal cystic areas, which showed evidence of variable sized blood products. On postcontrast MRI, there was central enhancement of these lesions (Figure 3) with intralesional traversing blood vessels (not shown). Prospective diagnosis based on MR findings was splenic peliosis with giant cavernous hemangioma being a close second possibility.

**Figure 4:**
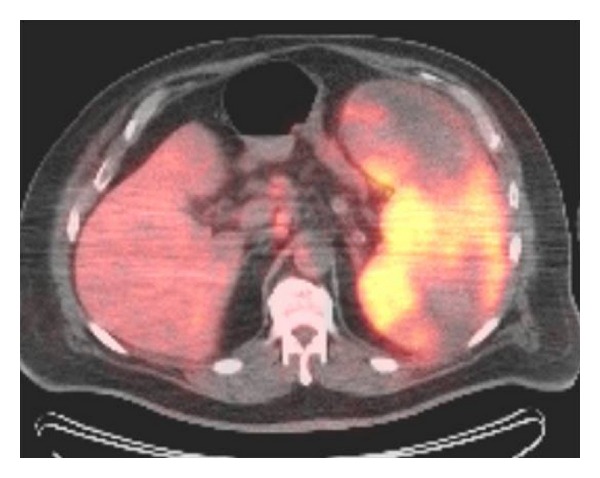
Axial PET CT image showing FDG uptake in the lesions suggestive of high metabolic activity.

 Bone marrow biopsy, however, revealed a B-cell lymphoma and an initial staging PET scan showed massively enlarged spleen with increased FDG uptake (Figure 4) with an SUV of 6.23 consistent with lymphoma or other malignant process [3, 6]. A week after the presentation, the patient presented in ER with splenic rupture and underwent emergent splenectomy, splenic pathology showed diffuse large B cell lymphoma.

## 3. Discussion

CT is the most widely used and currently the imaging modality of choice for assessment of lymphoma. The overall accuracy of CT in depicting splenic lymphoma is approximately 22–65% [3], differences between series may be partly explained by advances in multidetector imaging allowing greater spatial resolution and acquisition during optimal splenic enhancement. When focal splenic involvement is detected, deposits are of lower attenuation than adjacent normal splenic parenchyma on unenhanced CT and demonstrate little or no enhancement following injection of intravenous iodinated contrast medium [3, 7]. The lymphomatous deposits in spleen in our case were hyperdense on noncontrast CT with significant enhancement following administration of contrast.

Conventional T1- and T2-weighted MR sequences have poor sensitivity for splenic involvement in lymphoma, which is due to the similar relaxation times of lymphoma and normal splenic tissue using these sequences [8]. Identifiable foci within the spleen are hypointense and hyperintense on T1- and T2-weighted sequences, respectively. Gadolinium-enhanced MRI increases conspicuity of lymphoma deposits as a consequence of their relatively poor enhancement compared to normal splenic parenchyma [9, 10]. In our case, the MRI showed multifocal blood, filled cystic areas of variable age with marked enhancement postcontrast.

The differential diagnoses based on CT and MRI imaging characteristics involved pathologies with a high vascular content, namely, splenic peliosis, cavernous atypical hemangioma, or an angiosarcoma. The bone marrow biopsy obtained given the history of chemical exposure and the finding of pancytopenia, however, showed B cell lymphoma. This was confirmed by PET scan (SUV 6.23) and splenic pathology when patient presented with a splenic rupture. In a study of 68 patients with known malignant disease, an SUV threshold of 2.3 correctly differentiated benign from malignant lesions with the sensitivity, specificity, PPV, and NPV of 100%, 100%, 100%, and 100%, respectively [6].

In conclusion, splenic lymphoma could present with imaging features simulating a vascular malformation or tumor and should be considered in differential of a multifocal hypervascular lesion, in an appropriate clinical setting.
